# Anti-inflammatory Effects of a Novel Herbal Extract in the Muscle and Spinal Cord of an Amyotrophic Lateral Sclerosis Animal Model

**DOI:** 10.3389/fnins.2021.743705

**Published:** 2021-11-11

**Authors:** Sun Hwa Lee, Mudan Cai, Eun Jin Yang

**Affiliations:** ^1^Department of Clinical Research, Korea Institute of Oriental Medicine, Daejeon, South Korea; ^2^Department of Korea Medicine (KM) Science Research, Korea Institute of Oriental Medicine, Daejeon, South Korea

**Keywords:** amyotrophic lateral sclerosis, herbal medicine, oxidative stress, inflammation, skeletal muscle

## Abstract

Amyotrophic lateral sclerosis (ALS) is a complex disease characterized by motor neuron loss and muscle atrophy. There is no prominent treatment for ALS as the pathogenic process in the skeletal muscle and spinal cord is complex and multifactorial. Therefore, we investigated the effects of a herbal formula on the multi-target effects in the skeletal muscle and spinal cord in hSOD1^G93A^ transgenic mice. We prepared a herbal extract (HE) from *Glycyrrhiza uralensis*, *Atractylodes macrocephala Koidzumi*, *Panax ginseng*, and *Astragalus membranaceus*. Control and HE-treated mice underwent rotarod and footprint tests. We also performed immunohistochemical and Western blotting analyses to assess expression of inflammation-related and oxidative stress-related proteins in the muscle and spinal cord tissues. We found that the HE increased motor activity and reduced motor neuron loss in hSOD1^G93A^ mice. In addition, the HE significantly reduced the levels of inflammatory proteins and oxidative stress-related proteins in the skeletal muscles and spinal cord of hSOD1^G93A^ mice. Furthermore, we demonstrated that the HE regulated autophagy function and augmented neuromuscular junction in the muscle of hSOD1^G93A^ mice. Based on these results, we propose that the HE formula may be a potential therapeutic strategy for multi-target treatment in complex and multifactorial pathological diseases.

## Introduction

Amyotrophic lateral sclerosis (ALS) is a neurodegenerative disease that leads to progressive degeneration and death of the motor neurons and muscle paralysis. Ten percent of patients with ALS are known to have familial ALS that is caused by genetic mutations in genes, such as *SOD1* and *C9ORF72*. However, 90% of the cases are sporadic. Despite several attempts to find effective therapy, only two drugs are permitted for the treatment of ALS—riluzole reduces excessive glutamate excitotoxicity and edaravone reduces oxidative stress. However, even these show only a mild effect in delaying disease progression and extending life (Miller et al., 2012; Watanabe et al., 2018).

Amyotrophic lateral sclerosis is caused by the loss of motor neurons and muscle atrophy. However, it remains controversial whether muscle atrophy is caused by the loss of the motor neurons or pathogenic mechanisms in the muscle that lead to death of motor neurons. Wong and Martin (2010) demonstrated that expression of mutant human SOD1 (hSOD1) in the skeletal muscles causes motor neuron degeneration in an ALS animal model and suggested that the muscles could be the primary site for pathogenesis in ALS. Boillee et al. (2006) showed that microglia and astrocytes contribute to the degeneration of motor neurons, leading to disease pathogenesis. Therefore, therapeutic strategies should target both the motor neurons and skeletal muscles to alleviate the disease suffering and to improve the quality of life of patients with ALS and their families.

Herbal medicines, which are common, complementary, alternative medicines, are used worldwide for the treatment of various diseases, such as cancer (Molassiotis et al., 2005), immune dysfunction diseases (Liu et al., 2018), and neurodegenerative diseases (Zhang et al., 2015; Jarrell et al., 2018) and muscle regeneration and energy metabolism in muscle (Tan et al., 2014; Go et al., 2020).

Based on herbal function, herbal combinations, with each herb having differing mechanisms of action, help increase the therapeutic efficacy. According to a previous study by our group, Bojungikgi-tang (BJIGT) has neuroprotective effects and delays the progression of disease in an ALS animal model (Cai et al., 2019). Therefore, we designed the present study to find a herbal formula in BJIGT that increases the anti-inflammatory and anti-oxidative effects in the ALS model and to examine combined herbal extracts (HEs), including *Glycyrrhiza uralensis*, *Atractylodes macrocephala Koidzumi*, *Panax ginseng*, and *Astragalus membranaceus*, in the skeletal muscles and spinal cord in the ALS animal model. *G. uralensis* is primarily used for its anti-inflammatory effects on gastric ulcers (Katakai et al., 2002), as well as its anti-allergic and neuroprotective effects (Kobayashi et al., 1995; Yu et al., 2008; Hasanein, 2011). *Atractylodes macrocephala Koidzumi* is also used in traditional medicine for its anti-inflammatory (Li et al., 2007) and antitumor effects (Kimura, 2006). *P. ginseng* has been widely used to improve the motor functions in spinal cord injury models (Kim et al., 2015), and its active compound, ginsenoside Re, has increased anti-inflammatory effects in hSOD1^G93A^ mice and LPS-induced BV2 microglial cells (Lee et al., 2012; Cai and Yang, 2016). *A. membranaceus* is reported to have bioactive compounds with immunomodulatory, anti-inflammatory, and antioxidant effects in diabetic nephropathy and heart disease (Fu et al., 2014). Additionally, *A. macrocephala* regulates the mitochondrial function and energy metabolism in C_2_C_12_ myotubes (Song et al., 2015).

In the present study, we performed behavioral tests, including rotarod test and foot printing, immunohistochemistry, and Western blotting, in hSOD1^G93A^ mice. We found that the herbal combination extracts improved motor activity and increased anti-neuroinflammation and anti-oxidation activity in the skeletal muscle (tibialis anterior and gastrocnemius) and spinal cord of hSOD1^G93A^ mice.

## Materials and Methods

### Animals

Hemizygous male hSOD1^G93A^ mice were used as the ALS model. They were purchased from the Jackson Laboratory (Bar Harbor, ME, United States). hSOD1^G93A^ B6SJL mice carry a mutation of a glycine-to-alanine at the 93rd codon of the cytosolic Cu/Zn superoxide dismutase gene and were maintained as described previously (Yang et al., 2010). They were handled in accordance with the United States National Institutes of Health guidelines (Bethesda, MD, United States). Animal experiments were approved by the Institutional Animal Care and Use Committee of the Korea Institute of Oriental Medicine (protocol number: 17-061). All mice were housed in Specific Pathogen Free (SPF) animal facility and acclimatized at a constant temperature (21 ± 3°C) and humidity (50 ± 10%) under a 12 h light/dark cycle with free access to water and stand chow *ad libitum*.

### Preparation of Herbal Extracts and Treatment

Medicinal herbs, such as *G. uralensis*, *A. macrocephala Koidzumi*, *P. ginseng*, and *A. membranaceu*, were purchased from Kwangmyungdang Medicinal Herbs Co. (Ulsan, South Korea). For water extraction of medicinal herbs, these four medicinal herbs were mixed in a 1:1:1:1 ratio. Mixed herbs were extracted with distilled water for 24 h at room temperature, filtered through Whatman filter paper, and concentrated under reduced pressure. The extracts were then freeze-dried to obtain a powdered extract. The extracts were stored at −20°C for further use. For the treatment of HEs, the powdered extract was dissolved in distilled water before use.

Twenty-four male mice were randomly divided into the following groups: non-transgenic mice (nTg) = 8, hSOD1^G93A^ transgenic mice (Tg) = 8, and HE-treated hSOD1^G93A^ transgenic mice (Tg-HE) = 8. HEs were administered once daily for 6 weeks as an oral dose of 1 mg/g, starting in 8-week-old hSOD1^G93A^ transgenic mice. The nTg and hSOD1^G93A^ transgenic mice were the controls and were treated with distilled water.

### Rotarod Test

The mice were trained for 2 weeks before the test. To measure motor coordination, each mouse was placed on the rotating rod (10 rpm), as described previously (Yang et al., 2010). Each mouse was tested three times, and the average time spent on the rod was determined for each group.

### Footprint Test

Footprint tests were performed the day before the mice were euthanized to determine the extent of muscle loosening (Filali et al., 2011; Mancuso et al., 2011). The hind paws of mice were painted with a non-toxic, water-soluble ink to pass through an alley that was 70 cm in width, 16 cm in length, and 6 cm in height. At least three attempts were made to obtain a clearly visible footprint.

### Tissue Preparation

The tibialis anterior (TA) and gastrocnemius (GC) muscles and the spinal cords of mice were collected from hSOD1^G93A^ mice. Mice tissue were homogenized in radioimmunoprecipitation assay (RIPA) lysis buffer [50 mM Tris–Cl pH 7.4, 1% NP-40, 0.1% sodium dodecyl sulfate (SDS), and 150 mM NaCl], containing a protease and phosphatase inhibitor cocktail (Thermo Fisher Scientific, Waltham, MA, United States). Homogenate was quantified using bicinchoninic acid assay kit (Pierce, IL, United States).

### Western Blotting Analysis

Total proteins (20 μg) were separated by SDS-polyacrylamide gel electrophoresis and transferred to polyvinylidene difluoride membrane for Western blotting. The membranes were blocked with 5% skim milk (Sigma) in Tris–buffered saline for 1 h at room temperature and then incubated with various primary antibodies overnight at 4°C: tumor necrosis factor (TNF)-α, cluster of differentiation 11b (CD11b), heme oxygenase (HO)-1, ferritin, and tubulin (all 1:1,000; Abcam, Cambridge, MA, United States); NAD(P)H quinone dehydrogenase 1 (NQO1), B-cell lymphoma-2 (Bcl-2)-associated X protein (BAX), transferrin, and actin (all 1:1,000; Santa Cruz Biotechnology, Santa Cruz, CA, United States); p62 and microtubule-associated protein 1A/1B light chain (LC) 3B (all 1:1,000; Cell Signaling Technology, Danvers, MA, United States); and glial fibrillary acidic protein (GFAP) (1:5,000; Agilent Technologies, Santa Clara, CA, United States). Further, the blots were probed with horseradish peroxidase-conjugated anti-mouse or anti-rabbit secondary antibodies (Santa Cruz Biotechnology) and visualized using SuperSignal West Femto Substrate Maximum Sensitivity Substrate (Thermo Fisher Scientific). A ChemiDoc image analyzer was used to detect immunoblotted bands (Bio-Rad, Hercules, CA, United States).

### Immunohistochemistry

For neuromuscular junction, the experiment was performed, as described previously (Cai et al., 2019). Briefly, the GC tissue was fixed with 4% paraformaldehyde and incubated in 20% sucrose for 24 h. The GC tissue was then embedded in optimal temperature cutting compound, and the sections were cryo-sectioned. For staining, the sections were incubated with α-bungarotoxin (1:500 dilution) for 2 h.

### Nissl Staining

The spinal cord tissues were fixed with 4% paraformaldehyde and paraffinized. Further, the tissue slices were gradually dehydrated in alcohol (70, 80, 90, and 100%) for 5 min with two changes and placed in xylene for 5 min with three changes. Then, the slices were stained with 0.1% cresyl violet (Sigma, St. Louis, MO, United States) for 5 min, washed three times in distilled water, and dehydrated two times in gradient alcohol (70, 80, 90, and 100%) for 5 min each. Finally, the slices were transferred and rinsed again in xylene for three times, 5 min each time, and covered with a coverslip using Histomount media and dried at room temperature. The following criteria were used to quantify motor neuron loss: neurons must be located in the ventral horn of spinal cord L4∼5, the diameter of soma should be more than 20 μm, and neurons should have a distinct nucleolus (Lance-Jones, 1982).

### Statistical Analyses

Data are presented as the mean ± standard error of the mean (SEM), where indicated. The experiments were performed at least three independently and analyzed using GraphPad Prism 9.0 (GraphPad Software, San Diego, CA, United States). Comparisons between each group were analyzed by one-way analysis of variance (ANOVA) followed by Bonferroni’s multiple comparison tests or Newman-Keuls test.

## Results

### Herbal Extract Improves Motor Function in hSOD1^G93A^ Mice

**FIGURE 1:**
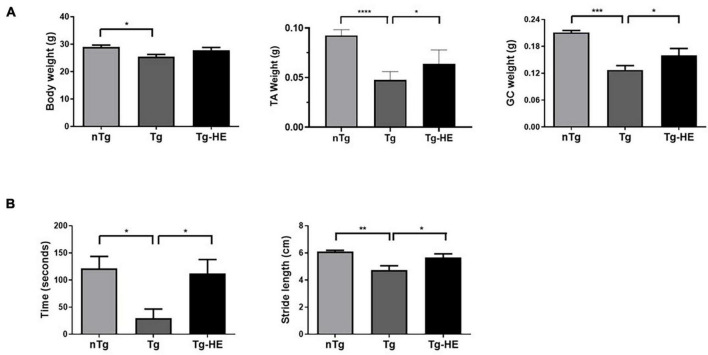
Effect of herbal extract (HE) on the motor functions in hSOD1^G93A^ mice. **(A)** The body weight and tibialis anterior (TA) or gastrocnemius (GC) muscle weight of non-transgenic mice, hSOD1^G93A^ mice, and HE-treated hSOD1^G93A^ mice. **(B)** Comparison of outcome of rotarod and footprint tests between groups. Data are expressed as the mean ± SEM (*N* = 8, *p < 0.05, **p < 0.01, ***p < 0.001, and ****p < 0.0001). The statistical analyses were conducted using one-way ANOVA followed by Bonferroni’s multiple comparison tests. nTg, non-transgenic mice; Tg, hSOD1^G93A^ mice; Tg-HE, HE-treated hSOD1^G93A^ mice.

The body weight and muscle weight of the mice were significantly reduced by 1.1- and 1.7-fold (*p* < 0.05, *p* < 0.001), respectively, in the Tg group compared with the nTg group (Figure 1A). There was no change in the body weight of mice in the Tg-HE group, although the muscle weights of TA and GC of mice in the Tg-HE group were significantly increased by 1.3- and 1.2-fold, respectively, compared with those of mice in the Tg group (Figure 1A).

The rotarod and footprint tests were performed as behavioral tests to determine the effect of HE administration on the motor function of the hSOD1^G93A^ mice after administration of the HE (1 g/kg) for 6 weeks. As shown in Figure 1B, compared with the motor function in the nTg group, motor function in the Tg group was reduced by 4.1-fold (*p* < 0.05) in the rotarod test. However, the Tg-HE group showed a 3.8-fold (*p* < 0.05) increase in motor function compared with the Tg group. Additionally, the Tg group showed stride length of 4.7 ± 0.3 cm, which was reduced by 1.3-fold (*p* < 0.01) compared with the nTg group; and the Tg-HE group showed stride length of 5.5 ± 0.2 cm, which was 1.2-fold (*p* < 0.05) that of the Tg group (Figure 1B).

### Herbal Extract Reduces Inflammation in the Muscles of hSOD1^G93A^ Mice

**FIGURE 2:**
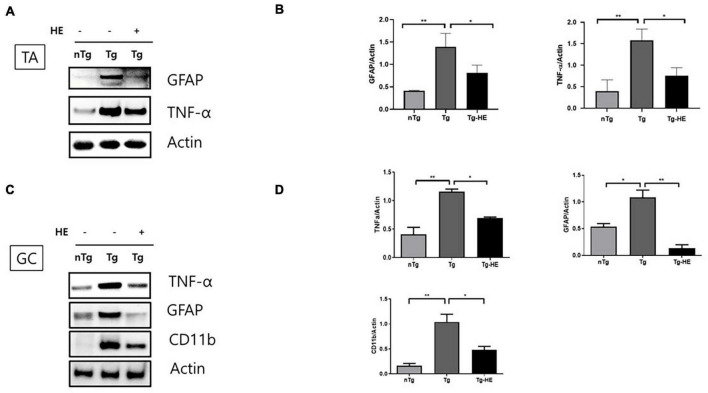
Herbal extract (HE) relieves inflammation in the tibialis anterior (TA) and gastrocnemius (GC) of hSOD1^G93A^ mice. **(A–D)** Western blotting of non-transgenic mice (nTg, *N* = 3), hSOD1^G93A^ mice (Tg, *N* = 3), and HE-treated hSOD1^G93A^ mice (Tg-HE, *N* = 3). **(A)** Data for Western blotting analysis of glial fibrillary acidic protein (GFAP) and tumor necrosis factor (TNF)-α in the TA. **(B)** Quantification of levels of GFAP and TNF-α with respect to levels of actin that is used as a loading control. **(C)** GC is immunoblotted with TNF-α, GFAP, and cluster of differentiation 11b (CD11b) using nTg, Tg, and Tg-HE (*N* = 3). **(D)** Quantification of levels of TNF-α, GFAP, and CD11b with respect to that of or actin that is used as a loading control. Data are shown as mean ± SEM. The statistical analyses were conducted with one-way ANOVA followed by Bonferroni’s multiple comparison tests (*p < 0.05 and **p < 0.01).

To examine the effect of HE on inflammation in the muscles of hSOD1^G93A^ mice, we investigated the expression of inflammation-related proteins—GFAP, TNF-α, and CD11b—by Western blotting. Quantitative analysis showed that the expressions of GFAP and TNF-α were increased by 3.3- and 4.6-fold (*p* < 0.01), respectively, in the TA of the Tg group compared with that in the nTg group (Figures 2A,B). However, treatment with HE significantly decreased their expressions by 1.7- and 2.0-fold (*p* < 0.05), respectively, in the Tg-HE group compared with the Tg group.

Moreover, the expressions of TNF-α, GFAP, and CD11b were increased by 2. 8−, 2. 0−, and 6.3-fold (*p* < 0.01, *p* < 0.05, and *p* < 0.01), respectively, in the GC of the Tg group compared with that in the nTg group; whereas treatment with HE significantly decreased expression of the same proteins by 1. 6−, 8. 0−, and 2.1-fold (*p* < 0.05, *p* < 0.01, and *p* < 0.05), respectively, in the Tg-HE group compared with the Tg group (Figures 2C,D).

### Herbal Extract Attenuates Oxidative Stress in the Muscles of hSOD1^G93A^ Mice

**FIGURE 3:**
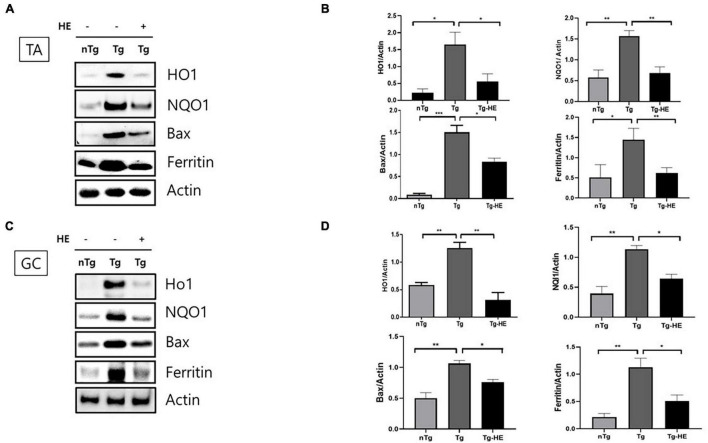
Herbal extract (HE) reduces oxidative stress in the muscles of hSOD1^G93A^ mice. Representative data for Western blotting analysis of HO1, NQO1, BAX, and ferritin in the **(A)** tibialis anterior (TA) and **(C)** gastrocnemius (GC) using nTg, Tg, and Tg-HE (*N* = 3); and quantitative analysis of the expression level of each protein in **(B)** TA and **(D)** GC. Data are shown as the mean ± SEM. The statistical analyses were conducted with a one-way ANOVA followed by Bonferroni’s multiple comparison tests (*p < 0.05, **p < 0.01, and ***p < 0.001).

We analyzed the effects of HE on the expression of oxidative stress-related proteins in the muscles of hSOD1^G93A^ mice. The expressions of HO1, NQO1, BAX, and ferritin in the TA of the Tg group increased by 7. 5−, 2. 6−, 7. 9−, and 2.8-fold (*p* < 0.05, *p* < 0.001, *p* < 0.001, and *p* < 0.05), respectively, compared with that in the nTg group. In contrast, in the GC, they were increased by 2. 1−, 2. 8−, 2. 1−, and 5.3-fold (*p* < 0.01, *p* < 0.01, *p* < 0.01, and *p* < 0.01), respectively, in the Tg group compared with the nTg group (Figures 3A–D). However, after administration of HE, expressions of HO1, NQO1, BAX, and ferritin in the TA significantly decreased by 2. 9−, 2. 2−, 2. 9−, and 2.3-fold (*p* < 0.05, *p* < 0.01, *p* < 0.05, and *p* < 0.01), respectively, in the Tg-HE group compared with the Tg group. On the other hand, they decreased in the GC by 4. 0−, 1. 7−, 1. 4−, and 2.2-fold (*p* < 0.05), respectively, in the Tg-HE group compared with that in the Tg group (Figures 3A–D).

### Herbal Extract Reduces the Expression of Autophagy-Associated Proteins in the Gastrocnemius of hSOD1^G93A^ Mice

**FIGURE 4:**
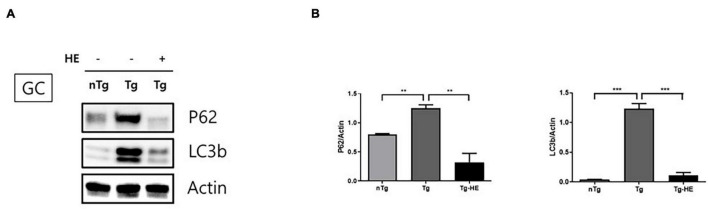
Herbal extract (HE) regulates autophagy dysfunction in the gastrocnemius (GC) of hSOD1^G93A^ mice. **(A)** GC is immunoblotted with p62 and LC3b using nTg, Tg, and Tg-HE (*N* = 3). **(B)** Protein expression is quantified relative to the expression of actin, which was used as loading control. Data are shown as the mean ± SEM. The statistical analyses were conducted with a one-way ANOVA followed by Bonferroni’s multiple comparison tests (**p < 0.01 and ***p < 0.001).

We investigated the expression of autophagy-related proteins, p62 and LC3B, to examine the effect of HE on autophagy dysfunction in the GC of hSOD1^G93A^ mice. The expression of p62 and LC3B increased by 1.5− and 28.0-fold (*p* < 0.05 and *p* < 0.001), respectively, in the GC in the Tg group compared with that in the nTg group (Figures 4A,B). However, treatment with HE significantly decreased their expression by 3.8− and 10.0-fold, respectively, in the Tg-HE group compared with that in the Tg group.

**FIGURE 5:**
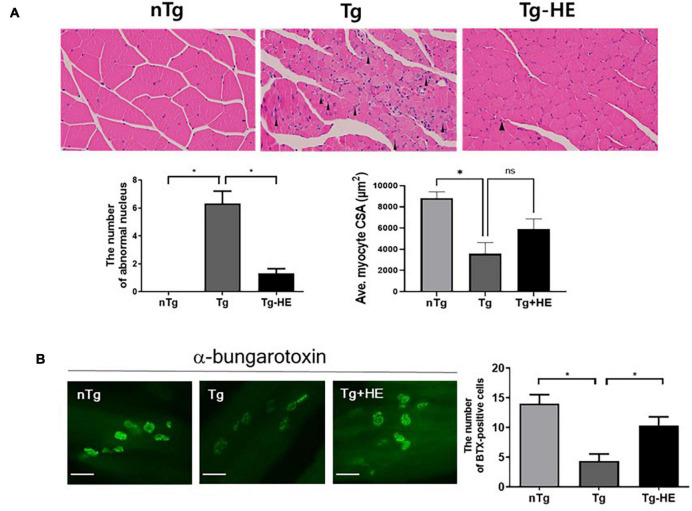
Herbal extract (HE) prevents muscle degeneration in the gastrocnemius (GC) of hSOD1^G93A^ mice. **(A)** Representative images of H&E staining of the GC of the nTg, Tg, and Tg-HE groups and quantification of the number of abnormal of nucleus and myocyte cross-sectional area (CSA). The arrowhead indicates an abnormal nucleus. **(B)** α-bungarotoxin staining of the GC and quantification of the number of BTX-positive cells (*N* = 3); scale bar = 500 μm. Data are shown as the mean ± SEM. The statistical analyses were conducted with a one-way ANOVA followed by Bonferroni’s multiple comparison tests (*p < 0.05).

In a previous study, we observed a relatively small muscle fiber diameter and abnormal muscle fiber nuclei in the skeletal muscles of hSOD1^G93A^ transgenic mice (Cai et al., 2015). To analyze the alterations in the structure of the GC muscle fiber following administration of HE, we performed hematoxylin/eosin staining of the cross sections of the muscle tissues. HE administration reduced the abnormal nuclei in GC by 4.7-fold and enlarged the fiber diameter compared to those of the Tg (Figure 5A). In addition, we found that enlarged the fiber diameter in GC of HE-treated group compared to those of the Tg but it was not significant (Figure 5A).

Next, we determined the effect of HE on the pathological morphology of the GC. We found that treatment with HE retarded the typical features of muscle atrophy, such as increases in the number of small muscle fibers and abnormal nuclei (Figure 5A). Additionally, α-bungarotoxin staining demonstrated that treatment with HE increased the number of neuromuscular junctions (NMJs) in the GC by 2.4-fold (*p* < 0.05) in the Tg-HE group compared with that in the Tg group (Figure 5B).

### Herbal Extract Reduces Expression of Neuroinflammation or Oxidative Stress-Related Proteins in the Spinal Cord of hSOD1^G93A^ Amyotrophic Lateral Sclerosis Mice

**FIGURE 6:**
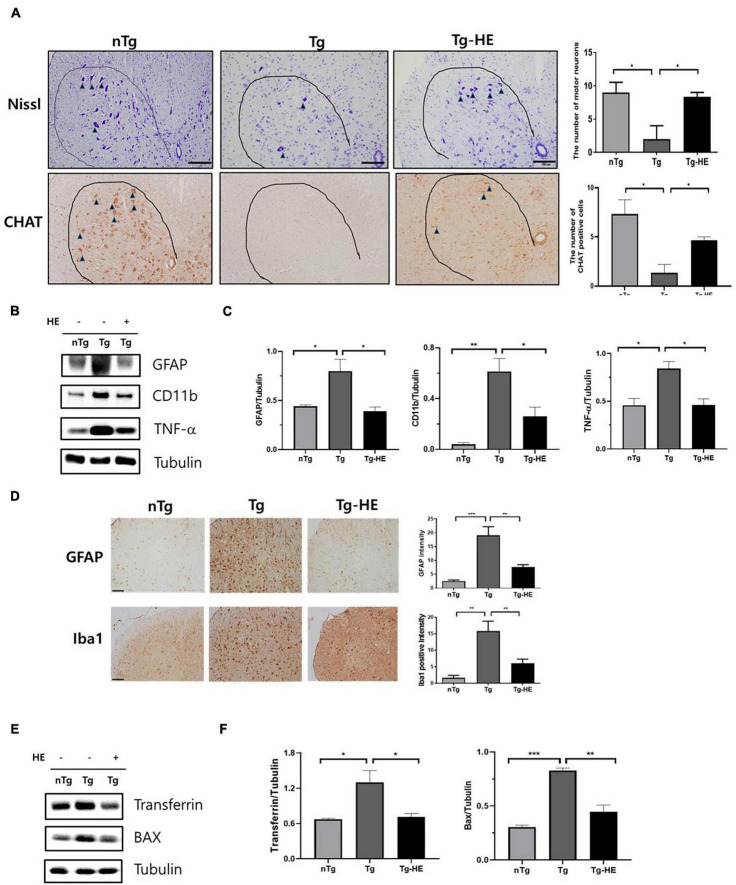
Herbal extract (HE) inhibits motor neuron death and increases anti-neuroinflammatory and anti-oxidative effects. **(A)** Nissl staining of the motor neurons (arrowheads) in the anterior horn of L4-5 lumbar spinal cords (*N* = 3); scale bar = 100 μm. **(B)** Representative Western blotting images showing the expression of inflammation-related proteins [glial fibrillary acidic protein (GFAP), cluster of differentiation 11b (CD11b), and tumor necrosis factor (TNF)-α] in the spinal cord of each group of mice (*N* = 3). **(C)** Quantification of immunoblots normalized to the expression of tubulin. **(D)** Immunohistochemistry with anti-GFAP and anti-Iba1 in spinal cord. scale bar = 2 mm. **(E)** Representative Western blotting images showing the expression of transferrin and BAX in the spinal cord of each group of mice (*N* = 3). **(F)** Quantification of immunoblots normalized to the expression of tubulin. Data are shown as the mean ± SEM. The statistical analyses were conducted with a one-way ANOVA followed by Newman-Keuls multiple or Bonferroni’s multiple comparison tests (*p < 0.05, **p < 0.01, and ***p < 0.001).

Neuroinflammation is a key cellular process in the pathogenesis of ALS (Liu and Wang, 2017). Nissl staining showed that the loss of motor neurons in the saline-treated Tg mice was dramatically reduced by 4.5-fold in the anterior horn of the lumbar spinal cord compared with that in the nTg mice (*p* < 0.05; Figure 6A). However, treatment with HE reduced the loss of motor neurons by 4.1-fold in the spinal cord of the hSOD1^G93A^ mice. This was consistent with the choline acetyltransferase immunohistochemistry data (*p* < 0.05; Figure 6A). To study the molecular mechanism of the motor neuron protection by HE, we investigated the levels of neuroinflammation-related proteins, such as GFAP, CD11b, and TNF-α, in the spinal cord of the hSOD1^G93A^ mice using Western blotting (Figures 6B,C). The analysis indicated that the expressions of GFAP, CD11b, and TNF-α were dramatically increased by 1. 8−, 15. 1−, and 1.8-fold (*p* < 0.05, *p* < 0.01, and *p* < 0.05), respectively, in the Tg group compared with in the nTg group; while treatment with the HE in the Tg-HE group significantly reduced the levels of GFAP, CD11b, and TNF-α by 2−, 2. 3−, and 1.8-fold (*p* < 0.05), respectively, compared with those in the Tg group (Figures 6B,C). In addition, we confirmed that HE treatment reduced by 2.4− and 2.5-fold (*p* < 0.01), respectively, the immunoreactivity of GFAP and Iba1 in anterior horn of the spinal cord compared to those of the Tg group (Figure 6D).

Oxidative stress is a mechanism contributing to ALS that leads to motor neuron cell death (Barber et al., 2006). To determine whether treatment with HE regulates oxidative stress, we evaluated the levels of oxidative stress-related proteins, transferrin and BAX, using Western blotting in the spinal cord of ALS mice (Figures 6E,F). The expression levels of transferrin and BAX were significantly increased by 1.9− and 2.7-fold (*p* < 0.05 and *p* < 0.001), respectively, in the Tg group compared with the nTg group, while treatment with HE in the Tg-HE group dramatically reduced their expression levels each by 1.8-fold (*p* < 0.05 and *p* < 0.01) compared with those in the Tg group.

## Discussion

Amyotrophic lateral sclerosis is a complex and incurable disease that leads to motor neuronal cell death and muscle paralysis. Studies have attempted to find treatment against ALS, but there is still no effective drug to treat ALS patients. Riluzole and edaravone have been used for the treatment of patients with ALS, but they do not offer complete cure. Therefore, it is necessary to develop drugs against multiple targets because ALS is a complex disease of the muscles and spinal cord.

Herbal medicine comprises multiple components and is primarily used to improve the immune system. Therefore, we investigated whether a herbal formula extract could be helpful in achieving immunity and anti-oxidation in the muscle and spinal cord of the hSOD1^G93A^ transgenic mice. The hSOD1^G93A^ transgenic mice have decreased muscle function in their skeletal muscles, such as strength, mitochondrial structure, and contractile apparatus, as well as loss of motor neurons in the spinal cord (Dobrowolny et al., 2008; Martin and Wong, 2020). Many studies have focused on the pathological mechanisms of motor neuron death (Renton et al., 2014; Alsultan et al., 2016; Taylor et al., 2016); however, only some studies have demonstrated that the skeletal muscles are a critical target for developing effective treatment for patients with ALS (Wong and Martin, 2010).

Oxidative stress is a critical factor leading to motor neuron death and muscle atrophy in ALS because oxidative stress impairs mitochondrial function and dysregulates protein homeostasis (Le Gall et al., 2020). Reactive oxygen species cause oxidative stress and increase production of cytokines and chemokines involved in abnormal glial oxidative responses in neurodegeneration (Nijssen et al., 2017). In the ALS model, immune cell infiltration is observed in the extensor digitorum longus muscle (Trias et al., 2018). Additionally, oxidative stress accelerates presynaptic decline in the NMJs and causes abnormal secretion of acetylcholinesterase. Therefore, the subsequent reduction in acetylcholine levels in the synaptic cleft can lead to the loss of muscle strength in patients with ALS (Pollari et al., 2014). Edaravone, an antioxidant, is a free radical scavenger and is used for the treatment of patients with ALS (Yoshino and Kimura, 2006), although it does not extend patients’ survival. Other antioxidants, including vitamin E, acetylcysteine, and creatine, were effective in ALS animal models, but they are not valuable for disease symptoms in patients with ALS (Louwerse et al., 1995). Therefore, studies on the discovery of multitarget treatment should be considered in ALS pathology as neuroinflammation and oxidative stress are linked and together causes loss of motor neurons and muscle degeneration. We focused on the anti-inflammatory and anti-oxidative effects of the herbal formula (*G. uralensis*, *A. macrocephala Koidzumi*, *P. ginseng*, and *A. membranaceus*) in the spinal cord and skeletal muscles of 14-week-old (presymptomatic stage) hSOD1^G93A^ mice to determine its preventive effect. We found that treatment with HE increased muscle weight and motor activity in the rotarod and foot printing tests. Additionally, we demonstrated that the expression levels of inflammation-related proteins (GFAP, TNF-α, and CD11b) and oxidative stress-related proteins (HO1, NQO1, Bax, and ferritin) were significantly reduced by HE in the TA and GC of hSOD1^G93A^ mice compared with those of the control mice.

Glial fibrillary acidic protein expression was increased in the hindlimb of GFAP-luc/SOD1^G93A^ mice at disease onset in damaged sciatic nerves ALS, suggesting that GFAP upregulation could be a valid marker at peripheral axons/neuromuscular junction and in the spinal cord/brain area according to the ALS pathogenesis stage (Keller et al., 2009).

Furthermore, we found that treatment with HE increased NMJ in the muscles to raise the motor function in hSOD1^G93A^ mice. These findings suggest that treatment with HE can delay disease progression through anti-inflammatory and anti-oxidative effects in the skeletal muscles of mice with ALS. However, whether HE can extend the survival of hSOD1^G93A^ mice remains to be investigated.

Mitochondrial dysfunction is detected in the muscles and spinal cord of hSOD1^G93A^ mice (Mattiazzi et al., 2002; Scaricamazza et al., 2020), plays a critical role in the degeneration of motor neurons in ALS, and is considered a therapeutic target due to its involvement in disease onset. Oxidative stress modulates the autophagy signaling pathway in the muscles and motor neurons (Rodney et al., 2016; Le Gall et al., 2020). Olivan et al. (2015) demonstrated a significant activation of the autophagy marker, LC3-II/LC3-I, in the muscles derived from hSOD1^G93A^ mice (Olivan et al., 2015). Furthermore, Zhou et al. (2019) showed that oxidative stress, mitochondrial dysfunction, and reduction in autophagy function promoted recurrent mitochondrial damage. Therefore, we suggest that an increase in motor activity and NMJ following treatment with HE results from loss of mitochondrial damage by anti-oxidation and autophagy regulation in hSOD1^G93A^ mice. As fiber transition from glycolysis to β-oxidation in the ALS muscle correlates with disease onset and defects in motor functions (Dobrowolny et al., 2018; Scaricamazza et al., 2020), the relationship between metabolic changes and motor function after treatment with HE should be investigated.

## Conclusion

This study demonstrated that treatment with HE improved motor activity and prevented the loss of motor neurons in hSOD1^G93A^ mice. Additionally, we identified anti-inflammatory and anti-oxidative mechanisms of HE in the skeletal muscles and spinal cord of ALS mice. Treatment with HE reduced the expression levels of inflammation-related proteins (GFAP, CD11b, and TNF-α) and oxidative stress-related proteins (HO1, NQO1, Bax, and ferritin) in the muscles (GC and TA) and spinal cord of hSOD1^G93A^ mice. The limitation is that we did not investigate the bio-active components of the anti-inflammatory and anti-oxidative effects of HE in the ALS model. Taken together, HE can be helpful for the treatment of multi-target complex diseases, such as ALS.

## Data Availability Statement

The raw data supporting the conclusions of this article will be made available by the authors, without undue reservation.

## Ethics Statement

The animal study was reviewed and approved by the Institutional Animal Care and Use Committee of the Korea Institute of Oriental Medicine (protocol number: 17-061).

## Author Contributions

SL performed the rotarod test, western blotting, and immunohistochemistry with the muscles, and partially wrote the manuscript. MC contributed to the foot printing test, western blotting, and immunohistochemistry with the spinal cord. EY designed the study, analyzed the data, and prepared the final version of the manuscript. All authors contributed to the article and approved the submitted version.

## Conflict of Interest

The authors declare that the research was conducted in the absence of any commercial or financial relationships that could be construed as a potential conflict of interest.

## Publisher’s Note

All claims expressed in this article are solely those of the authors and do not necessarily represent those of their affiliated organizations, or those of the publisher, the editors and the reviewers. Any product that may be evaluated in this article, or claim that may be made by its manufacturer, is not guaranteed or endorsed by the publisher.
